# Racecadotril Versus Loperamide for Acute Diarrhea of Infectious Origin in Adults: A Systematic Review and Meta‐Analysis

**DOI:** 10.1002/hsr2.70849

**Published:** 2025-05-22

**Authors:** Maryam Aziz, Laiba Urooj Malik, Erum Javed, Syeda Zuha Sami, Muhammad Maaz, Muhammad Arham Khan, Muhammad Farmaan, Omer Farooq, Umer Iqbal, Aashish Kumar, Syed Ali Arsal, Shafin Bin Amin, Inibehe Ime Okon

**Affiliations:** ^1^ Department of Medicine Dow University of Health Sciences Karachi Pakistan; ^2^ Department of Medicine Shaheed Mohtarma Benazir Bhutto Medical College Karachi Pakistan; ^3^ Department of Medicine Karachi Medical and Dental College Karachi Pakistan; ^4^ Department of Medicine, Khyber Medical College University of Peshawar Peshawar Pakistan; ^5^ Department of Medicine Jinnah Sindh Medical University Karachi Pakistan; ^6^ Department of Research Medical Research Circle (MedReC) Bukavu DR Congo

**Keywords:** diarrhea, infectious origin, loperamide, meta‐analysis, racecadotril, randomized controlled trials

## Abstract

**Background and Aims:**

Loperamide and racecadotril are two antidiarrheal medications with different mechanisms of action that are highly important for the treatment of diarrhea as a result of infectious pathology. Acute infectious diarrhea has a profound impact on our surroundings because of its detrimental effects on individual health. Medication such as racecadotril, among various other drug classes, plays a pivotal role in treating these diseases via the management of symptoms through its antisecretory, proabsorptive effects on the intestinal tract. The main objective of this analysis was to evaluate and contrast the usefulness and safety profiles of these drugs by combining information from randomized controlled trials and highlighting important side effects reported, such as constipation and stomach discomfort.

**Method:**

A total of 117 records were found after a thorough literature search was carried out across several databases, including PubMed, Google Scholar, and the Cochrane Library. The Cochrane Collaboration technique was utilized to evaluate the potential for bias. RevMan 5.2 data analysis was performed via a random effects model. The results are displayed as the mean difference (MD) with 95% CI for continuous data and relative risk (RR) with 95% confidence intervals (CIs) for dichotomous data. Statistical significance was established at *p* < 0.05.

**Results:**

Compared with loperamide, racecadotril significantly improved the clinical response (relative risk [RR] and 95% confidence interval [CI]). Additionally, the analysis of secondary outcomes revealed varying effects on abdominal pain, constipation, and abdominal enlargement, with moderate heterogeneity observed (*I*² = 56%).

**Conclusion:**

Compared with loperamide, racecadotril is a better therapeutic option for adult diarrhea caused by infection.

## 1 Introduction

Diarrhea is a prevalent condition worldwide that can be characterized by a minimum frequency of three episodes per day. These episodes may be accompanied by additional symptoms such as stomach discomfort, cramping, and a feeling of urgency [1]. The main etiologic factors pertaining to diarrhea can be of infectious or noninfectious origin. Bacteria, viruses, and other microorganisms lead to infectious diarrhea, while noninfectious diarrhea can have multiple causes [2].

Compared with any other gastrointestinal disease, infectious diarrhea is a leading cause of death globally. Approximately 1.6 million deaths were reported in 2016 alone, making infectious diarrhea the eighth leading cause of death worldwide [3]. Within the United States alone, nearly 179 million cases of diarrhea have been estimated according to the latest reports [4].

Antidiarrheal drugs are differentiated on the basis of their mechanisms of action, such as reducing motility, decreasing the secretions of the intestines, and increasing the time of absorption [5, 6]. Common antidiarrheal drugs include loperamide, diphenoxylate‐atropine, racecadotril, octreotide, clonidine, pancreatic enzymes, 5‐HT3 receptor antagonists, diosmectite, cholestyramine, probiotics, and rifaximin, among others [5, 6, 7, 8]. The most common and frequently used drug to treat diarrhea is loperamide [6].

While taking care of diarrhea, antidiarrheal drugs can also have side effects depending on their usage [9]. Common symptoms of antidiarrheal medicines are severe constipation, abdominal pain, bloating, nausea, and vomiting [10]. Another article reported hypotension as an adverse effect [11]. Therefore, it is crucial to be aware of the potential effects while using these medications [9]. Our meta‐analysis aimed to combine different studies comparing the efficacy of the antidiarrheal drugs racecadotril and loperamide. Our study also focused on different secondary outcomes of both drugs, such as abdominal pain, distention, enlargement, and constipation.

## 2 Methodology

### 2.1 Eligibility Criteria

Only randomized controlled trials were included in this meta‐analysis. They looked at the effects of loperamide and racecadotril on patients with an infectious source of diarrhea. The meta‐analysis included only RCTs that focused on acute infectious diarrhea, defined as diarrhea lasting less than 14 days. This ensured that the analysis excluded chronic forms of diarrhea, which can persist longer than 14 days. RCTs whose intervention group was administered racecadotril were selected, whereas the control group used loperamide among those who had diarrhea from infections. The research involved adult patients, ranging in age from young adults to the elderly. Separate analyses of pediatric populations were not performed, as all the included studies involved adult participants aged 18 years and older till 96 years. Qualitative articles, nonhuman studies, letters to editors/experts, and abstracts presented by conferences were not considered for analysis because they did not address the study question directly or provide data that could be retrieved for this review. After all, such studies lack a comparison group for comparison with other forms of reviews written in English. Furthermore, strict adherence to RCTs was done to evade any biases in the meta‐analysis, as well as ensuring a consistent analytical approach was carried out for all studies. Even though the concept of other study types could bring enhancement from a singular point of view, using RCTs specifically calibrates more the preciseness of the meta‐analysis by distributing confounding variables equally through randomization, as well as providing a controlled environment to better engage the direct assessment of the treatment groups throughout the study and thus only studies following the randomized clinical trial design were accepted.

### 2.2 Literature Search and Search Strategy

A thorough search strategy was developed using keywords such as “racecadotril” OR “enkephalinase,” “loperamide” OR “antidiarrheal,” and “acute diarrhea” because our focus was on finding information. We looked for original research comparing loperamide with racecadotril in patients with infectious diarrhea by searching electronic databases such as PubMed, Cochrane, and Google Scholar. These keywords produced 117 results. Furthermore, a manual search was conducted through the reference lists of the included articles to identify other relevant studies. Conversely, this study was not limited to any linguistic factors. This study adhered to the Preferred Reporting Items for Systematic Reviews and Meta‐Analyses (PRISMA) guidelines, which ensure transparency and accuracy in the review process, and its protocol is registered with PROSPERO.

### 2.3 Study Selection

The first step in the study selection process was identifying possibly relevant studies by analyzing the titles and abstracts of the accumulated data. Following a study of the selected articles' entire texts, two researchers assessed each to decide whether they might include it in the meta‐analysis. Studies comparing the efficacy of loperamide with that of racecadotril in treating individuals with infectious diarrhea, particularly in the adult population, have been conducted. Excluded studies included racecadotril and loperamide in combination with other medications or placebos. When differences surfaced, discussions with a third researcher were held to reach an agreement. To improve transparency in the review process, the PRISMA flowchart (Figure 2) was used to demonstrate the research selection process and the justifications for omitting studies. This comprehensive approach uses an unbiased and thorough methodology.

### 2.4 Data Extraction

The data extraction process was independently conducted by two investigators to ensure accuracy, with a subsequent review by a third investigator for validation. Our analysis included baseline information, intervention details, patient numbers, dichotomous data, and continuous data from the selected randomized controlled trials (RCTs) as well as from other RCTs. The study's primary outcome was the clinical response of each treatment group, being the time from the first dose of treatment to the resolution of diarrhea or the production of the first formed stool. Both sets of data were dichotomous. Constipation, abdominal enlargement/distension, and abdominal pain were among the secondary outcomes. These outcomes are analyzed via odds ratios. The study title, publication year, primary author's name, study year, country of origin, participant count, type of intervention used, and outcome measures assessed were all recorded in this comprehensive data collection form. Furthermore, the form included detailed trial characteristics, including author details, country of origin, DOI, journal, year of publication, type of study, age in the intervention group, age in the placebo group, number of participants in the intervention group, number of participants in the placebo group, and gender distribution (percentage of males) in both groups.

### 2.5 Risk of Bias Assessment

**Figure 1:**
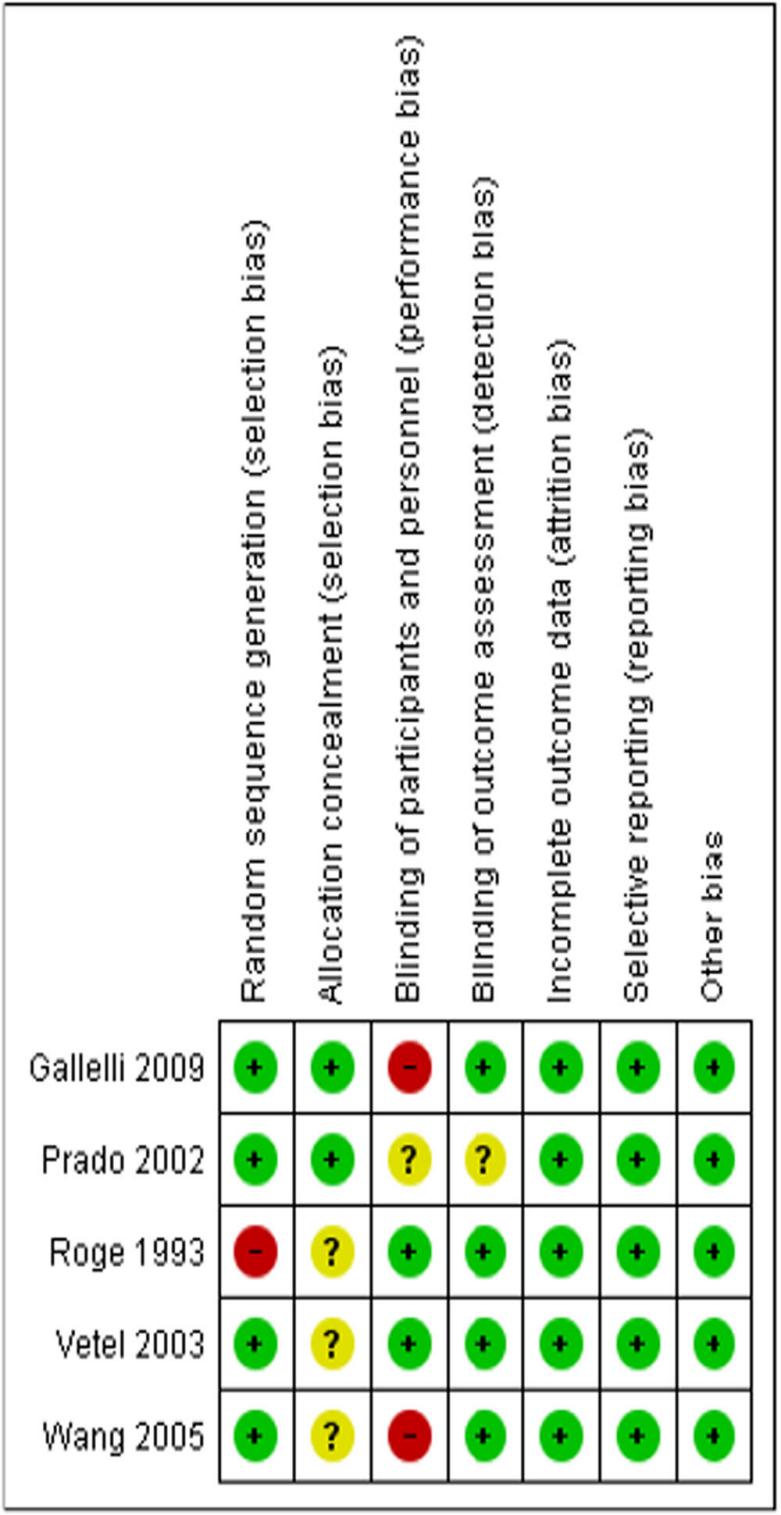
Risk of bias summary: Review authors' judgments about every risk of bias item for each included study.

To formally evaluate the quality of the selected studies, two authors were chosen, and they used the Cochrane Collaboration method for assessing the risk of bias in randomized studies. Essential factors such as participant blinding, random sequence generation, outcome assessment, incomplete outcome data, and more potential causes of bias were taken into consideration when evaluating bias. On the basis of their assessment, the studies were categorized into three groups: unclear risk of bias due to incomplete data, high risk of bias, and low risk of bias. When there were differences, a third researcher was involved in the discussions to obtain a consensus. The risk of bias summary and graphical assessment is detailed in Figure 1 and see Supporting Information S1: Figure S1 respectively.

## 3 Statistical Analysis

The data analysis was performed via RevMan 5.2 (Review Manager, version 5.2). A random‐effects model was used to determine the outcomes of interest in this meta‐analysis. For dichotomous data, findings are presented as the relative risk (RR) (define as; a measure of the risk of a certain event happening in one group compared to the risk of the same event happening in another group.) and 95% confidence interval (CI) (define as; a range of values that you can be 95% certain contains the true mean of the population). For continuous data, we used the mean difference (MD) (defined as a statistical measurement that compares the average values of two groups) and corresponding 95% CI(s). A *p* value < 0.05 was considered a statistically significant result; statistical heterogeneity was visually evaluated via a forest plot via the *χ* ^2^ test (define as; a statistical test used to compare observed results with expected results) and *I* ^2^ test (define as; a statistical measure used in meta‐analyses to assess study heterogeneity, or the amount of variation between studies). Heterogeneity was considered high when the *p* value was < 0.5 or *I* ^2^ > 50%, in which case a leave‐one‐out analysis was performed to exclude the cause, if any.

## 4 Results

### 4.1 Search and Selection Process

**Figure 2:**
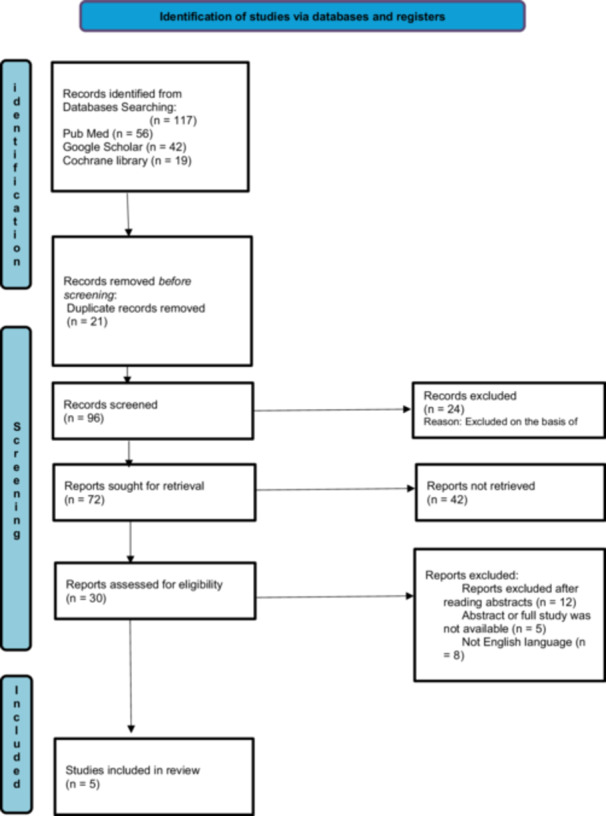
PRISMA flow chart.

In our meta‐analysis of five RCTs, we adhered to the PRISMA guidelines. Initially, we identified 117 records from PubMed (56), Google Scholar (42), and the Cochrane Library (19). After 21 duplicate records were removed, 96 records were screened, resulting in the exclusion of 24 records on the basis of title. We sought to retrieve 72 reports for full‐text assessment, of which 42 reports were not retrieved due to their inaccessibility pertaining upon trial by multiple authors even on multiple occasions throughout the study, in dire efforts to retrieve them, yet still led to failure; hence, 30 reports were assessed for eligibility. Ten reports were excluded after the abstracts were read, five were excluded because of the unavailability of full texts or abstracts, and eight were excluded because they were not in English. Any incongruities or discrepancies in judgments were resolved through discussion and agreement between the two authors. In addition, the reference lists of the included studies were also searched manually to extract any significant studies possibly missed during the initial search. Finally, five [12, 13, 14, 15, 16] studies satisfying the inclusion criteria were included in our meta‐analysis. The PRISMA flowchart below summarizes our screening process (Figure 2).

### 4.2 Study Characteristics

The comprehensive baseline characteristics of the studies included in our meta‐analysis can be reviewed in Table 1, which shows the effects of racecadotril compared with loperamide in patients with diarrhea of infectious origin. Details about the country, design of the study, interventional group, control group, patient characteristics and outcomes of the selected studies are also reported. Our sample included males and females whose mean age in the intervention group was between 36 and 90 years, and that in the placebo group was between 35 and 93 years. A total of five studies [12, 13, 14, 15, 16] consisting of 1294 participants, comprising males and females in the intervention and placebo groups, were included. It is of note that only Gallelli et al. [13] performed stool cultures, confirming the absence of bacterial pathogens in the study population and other studies, such as Prado [16] and Vetel et al. [15], presumed an infectious etiology but did not perform stool cultures. All the studies reported diarrhea of watery type. Our study included participants with different outcomes, and the data were analyzed via RevMan.

**Table 1:** Characteristics of the included studies.

Study characteristics														
Article no.	Author	Year	Type of study	Country	Sample size		Age		Gender (*M*%)		Duration of diarrhea before treatment	Type of diarrhea	Stool cultures	Infectious etiology
					Racecadotril	Loperamide	Racecadotril	Loperamide	Racecadotril	Loperamide				
1	Wang [12]	2005	Two‐center, randomized, parallel‐group, single‐blind study.	China	31	31	38.4 ± 15.1	34.7 ± 12.3	48.4	54.8	< 5 days	Watery diarrhea	Not performed	Presumed infectious
2	Gallelli et al. [13]	2009	Prospective, randomized, double‐blind trial.	Italy	30	31	83.9 ± 17	82.6 ± 14.8	30	32.26	Not reported	Watery diarrhea	Performed	Nonbacterial (negative cultures)
3	Roge et al. [14]	1993	Double‐blind, controlled clinical trial	France	37	32	40.8 ± 2.9	44.2 ± 3.3	44.4	41.2	< 5 days	Watery diarrhea	Not performed	Presumed infectious
4	Vettel et al. [15]	2003	Randomized, double‐blind, double‐placebo, parallel‐group study	France	82	75	40.9 + 1.8	41.5 + 2.2	33	33	Not reported	Watery diarrhea	Not performed	Presumed bacterial
5	Prado [16]	2002	Single‐blind, randomized, comparative, parallel‐group, multinational, multicenter study	Guatemala	473	472	35.9 ± 12.1	36.4 ± 13.5	57	50	2.1 days	Watery diarrhea	Not performed	Presumed bacterial

#### 4.2.1 Analysis of Outcomes

According to our PICO criteria, the outcomes of our study were the effects of racecadotril compared with loperamide in patients with diarrhea of infectious origin. However not all studies could be included for every outcome as they came short of providing data appropriate enough to warrant them included in those outcomes, this arose from inconsistencies in the type of outcomes reported throughout each study.

#### 4.2.2 Primary Outcomes

##### 4.2.2.1 Treatment Response by Clinical Group

**Figure 3:**
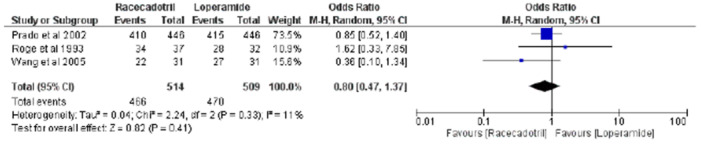
Forest plot of the clinical response to racecadotril vs. loperamide.

During the meta‐analysis of our study, we analyzed three studies [12, 14, 16] to evaluate the possibility of a clinical response. The overall pooled effect of analysis of three studies showed an odds ratio of 0.80 [95% CI (0.47, 1.37), *χ* ^2^ = 2.24, *p* = 0.41, *I* ^2^ = 11%], explaining the nonsignificant results, as shown by a *Z* value of 0.82 (*p* = 0.41), indicating that the racecadotril group responded more strongly to treatment than did the loperamide group. Moreover, low heterogeneity was also observed, which refers to variation in the study outcomes between studies. For publication bias, a funnel plot (see Supporting Information S1: Figure S2) can be constructed, which is symmetrical and predicts low chances of bias. Figure 3 shows the forest plot, which explains the OR and 95% CI for the effect of racecadotril versus loperamide.

#### 4.2.3 Secondary Outcomes

##### 4.2.3.1 Constipation

During the meta‐analysis of our study, we analyzed four studies [12, 13, 14, 16] to analyze the occurrence of therapy emergent constipation. The overall pooled effect analysis of the four studies revealed an odds ratio of 0.21 [95% CI (0. 13, 0.33), *χ* ^2^ = 0.99, *p* < 0.001, *I* ^2^ = 0%], revealing significant results, as shown by a *Z* value of 8.60 (*p* < 0.001), indicating that the racecadotril group had a lower incidence of therapy emergent constipation than the loperamide group did. For publication bias, a funnel plot (see Supporting Information S1: Figure S3‐A) was constructed, which was symmetrical and predicted moderate chances of bias. Supporting Information S1: Figure S3‐B shows the forest plot, which explains the OR and its 95% CI regarding the effect of racecadotril versus loperamide.

##### 4.2.3.2 Abdominal Pain

For the meta‐analysis of our study, we analyzed two studies [12, 16] to assess the incidence of abdominal pain. The overall pooled effect of the two studies yielded an odds ratio of 0.37 [95% CI (0.02, 6.43), *χ* ^2^ = 2.30, *p* = 0.49, *I* ^2^ = 56%], indicating that the results were nonsignificant and that the racecadotril group was more likely than the loperamide group to experience abdominal pain in their patients. Additionally, the heterogeneity test performed regarding the variation in study outcomes between studies revealed (*χ* ^2^ = 2.30, df = 1, *p* = 0.13), indicating high heterogeneity (*I* ^2^ = 56%). However, owing to high heterogeneity, we could not perform sensitivity analysis to assess the risk of bias by the leave‐one‐out method because few studies have reported the incidence of abdominal pain, which could introduce bias in the results. For publication bias, a funnel plot (see Supporting Information S1: Figure S4‐A) was constructed, which was symmetrical and predicted moderate chances of bias. Supporting Information S1: Figure S4‐B shows the forest plot, which explains the OR and its 95% CI regarding the effect of racecadotril versus loperamide.

##### 4.2.3.3 Enlarged Abdomen

In the meta‐analysis of our study, we analyzed two studies [15, 16] to assess the occurrence of abdominal enlargement. The overall pooled effect of analysis of three studies presented an odds ratio of 0.53 [95% CI (0.13, 2.16), *χ* ^2^ = 6.17, *p* = 0.38, *I* ^2^ = 84%], presenting nonsignificant results, indicating that racecadotril group showed a beneficial response as compared to the loperamide group. Additionally, the heterogeneity test performed regarding the variation in study outcomes between studies (*χ* ^2^ = 6.17, df = 1, and *p* = 0.01) revealed high heterogeneity (*I* ^2^ = 84%), but sensitivity analysis could not be performed due to insufficient studies showing abdominal enlargement. For publication bias, a funnel plot (see Supporting Information S1: Figure S5‐A) was generated. Supporting Information S1: Figure S5‐B shows the forest plot, which explains the OR and its 95% CI regarding the effect of racecadotril versus loperamide.

## 5 Discussion

Two oral medications, racecadotril and loperamide, were compared through this meta‐analysis to analyze the safety and effectiveness of their use in treating acute diarrhea of infectious pathology in the adult population. Racecadotril, also known as acetorphan, functions as an antisecretory agent in the gastrointestinal wall. Its impact is produced by blocking the enzyme enkephalinase located on the lining of the intestines. Through the lysis of enkephalin, this enzyme decreases the absorption of gut contents, resulting in increased secretions. Thus, the effects of racecadotril occur through the inhibition of the enzyme enkephalinase, which prevents the breakdown of Enkephalin and increases the absorption of the gut contents, aiding in the treatment of diarrhea [17].

Loperamide, which is sold commonly under the trade name “Imodium”, has a slightly different mechanism to decrease the frequency of diarrheal episodes. It binds to the mu‐opioid receptor in the gut wall. Through this interaction, it decreases gut movements and allows increased absorption of fluid and electrolytes through the intestinal passage, thus reducing stool frequency [18].

Several studies have been conducted, highlighting the contrasting features between racecadotril and loperamide, despite being an alternative treatment for the same pathologic condition. A significant number of trials have also been conducted to evaluate individual responses to these medications alongside adverse effects, including nausea, vomiting, abdominal discomfort, and any additional undesired reactions. Clinical studies have shown that racecadotril does not have a significant effect on gut motility. This mechanism can be particularly helpful when treating an infectious diarrheal condition, as normal gut movements are required to eradicate the pathogen. On the other hand, loperamide works by increasing the bulk of the stool and reducing the frequency and volume of diarrhea. Although these effects are useful, through their ability to reduce gut motility, several adverse events, such as therapy emergent constipation or toxic megacolon in complicated cases, have also been reported [19].

A study conducted for the effects of racecadotril highlighted that while both medications can effectively reduce the frequency of diarrheal episodes, racecadotril has a significant advantage in that it does not affect normal bowel function [20]. Multiple studies have shown that the side effects associated with racecadotril, such as nausea, vomiting, and abdominal pain, are mild compared with the severe adverse events reported with the use of loperamide [21, 22]. However, it is of note that in the study by Prado [16], racecadotril resolved the symptoms of acute diarrhea rapidly and effectively and produced more rapid resolution of abdominal symptoms and less constipation than loperamide. Also of mention should be the findings of Wang [12] as these two different medications show similar adverse events such as constipation, bloody stool, abdominal pain, skin itching, palpitation, dizziness, cold sweating, and headache. Racecadotril shows further benefits in terms of clinical response, such as a reduced incidence of therapy‐emergent constipation, decreased stool volume within a day of treatment onset, and a rapid reduction in abdominal distention and the severity of symptoms [14, 23]. The meta‐analysis specifically focused on adult populations to ensure homogeneity and relevance of the findings, as the included RCTs enrolled participants aged 18–96 years. Concerning another aspect of the results found in the conducted meta‐analysis another noteworthy point should be the age differences in the adult population which ranged from very young adults to older individuals and even though these age‐related differences in response to treatments may exist, stratified analyses within the included studies allow for insights into the efficacy and safety of racecadotril and loperamide across younger and older adult subsets. Notably, the elderly population often presents with distinct physiological characteristics, such as altered gut motility and comorbid conditions, which could influence drug efficacy and safety profiles. While a study [13] highlighted these distinctions, the clinical response trends observed across studies were consistent, supporting the generalizability of findings within the adult cohort. Future research with larger, age‐specific subgroups may provide further clarity, but for the purposes of this analysis, the inclusion of adult patients from diverse age ranges ensures robust and clinically meaningful conclusions. Overall, racecadotril has shown better tolerability than loperamide does and may be regarded as a better treatment plan for patients at risk of side effects [24, 25].

## 6 Limitations and Publication Bias

Notably, some limitations existed in our meta‐analysis, including the limited number of studies we found for a few outcomes, including abdominal pain and enlargement, which impeded the ability to perform a sensitivity analysis and increased the risk of bias. Despite this data, the funnel plots generated for publication bias seem symmetrical, indicating that publication bias seems unlikely to have a profound effect on our findings.

## 7 Conclusion

In conclusion, although both racecadotril and loperamide showed comparable efficacy in terms of our primary outcomes of clinical response, racecadotril was associated with a more significant improvement in the reduction of therapy‐emergent constipation. However, these patients might have an increased risk of abdominal pain and enlargement, although the findings were insufficient for a definitive presentation. Clinicians should weigh these results according to individual patient profiles when treating patients with acute diarrhea of infectious origin. Further studies with larger sample sizes and more consistent findings are needed to confirm and weigh the advantages and disadvantages of these treatments on the observed outcomes.

## Author Contributions

**Maryam Aziz:** conceptualization, methodology, data curation, investigation, validation, writing – original draft, writing – review and editing, project administration, supervision, resources, formal analysis. **Laiba Urooj Malik:** conceptualization, investigation, methodology, validation, writing – original draft, writing – review and editing, formal analysis, project administration, data curation, resources. **Erum Javed:** conceptualization, investigation, methodology, validation, writing – original draft, writing – review and editing, formal analysis, project administration, resources, data curation. **Syeda Zuha Sami:** conceptualization, investigation, writing – original draft, writing – review and editing, validation, methodology, formal analysis, project administration, data curation, resources. **Muhammad Maaz:** conceptualization, investigation, writing – original draft, methodology, validation, writing – review and editing, formal analysis, project administration, data curation, resources. **Muhammad Arham Khan:** conceptualization, investigation, writing – original draft, methodology, validation, writing – review and editing, formal analysis, project administration, data curation, resources. **Muhammad Farmaan:** conceptualization, investigation, writing – original draft, methodology, validation, writing – review and editing, formal analysis, project administration, data curation, resources. **Omer Farooq:** conceptualization, investigation, writing – original draft, methodology, validation, writing – review and editing, formal analysis, project administration, data curation, resources. **Umer Iqbal:** conceptualization, investigation, writing – original draft, writing – review and editing, methodology, validation, formal analysis, project administration, data curation, resources, supervision. **Aashish Kumar:** conceptualization, investigation, writing – original draft, methodology, validation, writing – review and editing, formal analysis, project administration, data curation, resources. **Syed Ali Arsal:** conceptualization, investigation, writing – original draft, writing – review and editing, methodology, validation, formal analysis, project administration, data curation, resources. **Shafin Bin Amin:** conceptualization, investigation, writing – original draft, methodology, validation, writing – review and editing, formal analysis, project administration, data curation, resources. **Inibehe Ime Okon:** investigation, funding acquisition, writing – review and editing, supervision.

## Ethics Statement

The authors have nothing to report.

## Consent

The authors have nothing to report.

## Conflicts of Interest

The authors declare no conflicts of interest.

## Transparency Statement

The corresponding author Inibehe Ime Okon affirms that this manuscript is an honest, accurate, and transparent account of the study being reported; that no important aspects of the study have been omitted; and that any discrepancies from the study as planned (and, if relevant, registered) have been explained.

## Supporting information

For review and publication.

## Data Availability

The authors have nothing to report.
