# Supplementary material for: Racecadotril Versus Loperamide for Acute Diarrhea of Infectious Origin in Adults: A Systematic Review and Meta‐Analysis
Source: Health Sci Rep. 2025 May 22;8(5):e70849. doi: 10.1002/hsr2.70849 (PMC12098968; doi:10.1002/hsr2.70849)

**For review and publication:**

**Supplementary Figure 1**. Risk of bias graph


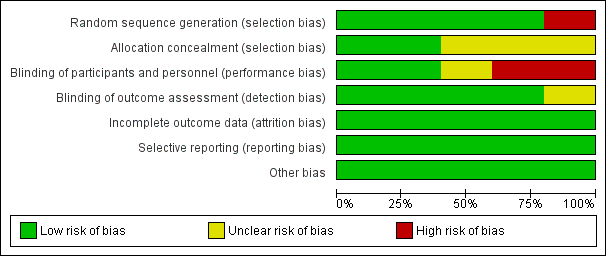


**Supplementary Figure 2.** Funnel plot of Clinical Group in Racecadotril vs loperamide


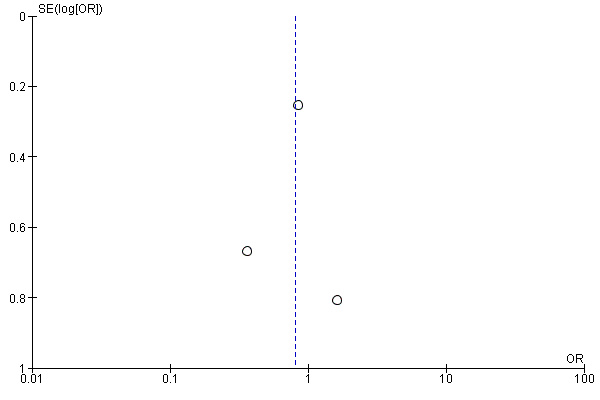


**Supplementary Figure 3-A.** Funnel plot of Constipation in Racecadotril vs loperamide

**
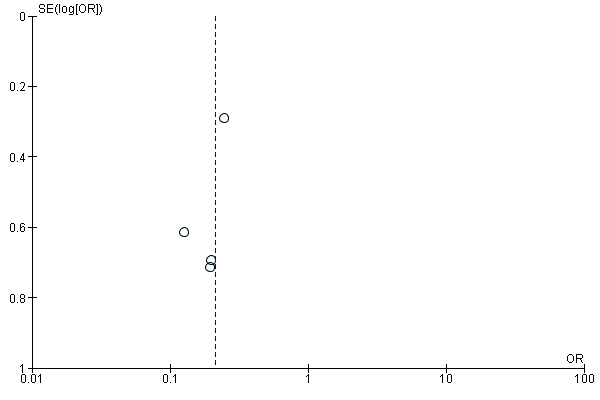
**

**Supplementary Figure 3-B.** Forest plot of Constipation in Racecadotril vs loperamide

**
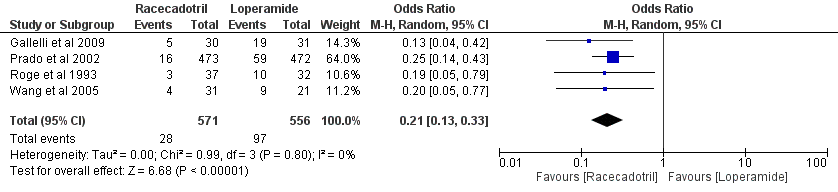
**

**Supplementary Figure 4-A.** Funnel plot of Abdominal Pain in Racecadotril vs loperamide


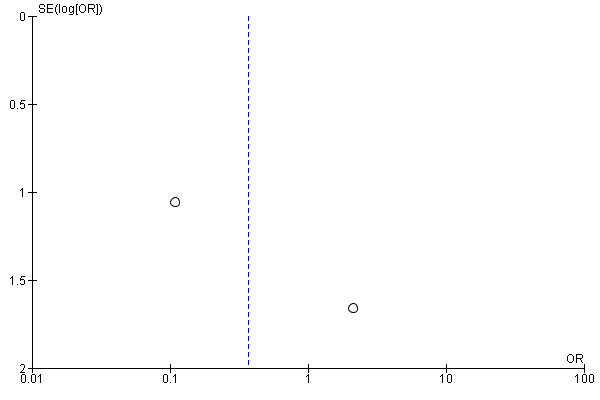


**Supplementary Figure 4-B.** Forest plot of Abdominal Pain in Racecadotril vs loperamide


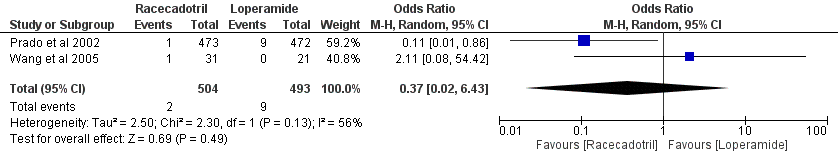


**Supplementary Figure 5-A.** Funnel plot of Enlarged Abdomen in Racecadotril vs loperamide
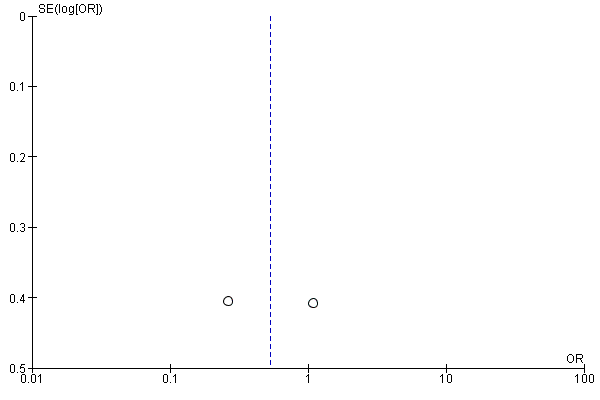


**Supplementary Figure 5-B.** Forest plot of Enlarged Abdomen in Racecadotril vs loperamide


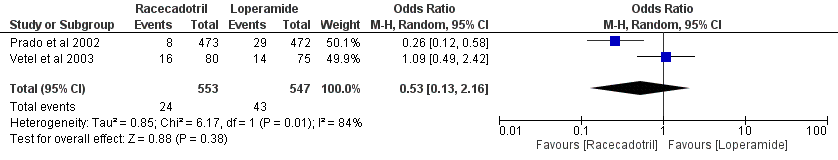

Supplement: Supplementary file 1 — For review and publication. [file HSR2-8-e70849-s001.docx]
